# “Give Me the Opportunity”: Mixed Ability Sports and Quality of Life in People with Intellectual Disabilities

**DOI:** 10.3390/sports13070208

**Published:** 2025-06-26

**Authors:** Pablo Elipe-Lorenzo, Miguel Ángel Verdugo, Pelayo Diez-Fernández, Brais Ruibal-Lista, Sergio López-García

**Affiliations:** 1 Faculty of Education, Pontifical University of Salamanca, 37007 Salamanca, Spain; pelipelo@upsa.es (P.E.-L.); slopezga@upsa.es (S.L.-G.); 2 Grupo de Investigación en Actividad Física, Deporte y Salud (GIADES), Facultad de Educación, Universidad Pontificia de Salamanca, 37007 Salamanca, Spain; brais.ruibal@frayluis.com; 3 INICO-Faculty of Psychology, University of Salamanca, 37005 Salamanca, Spain; verdugo@usal.es; 4 EUM Fray Luis de León, Catholic University of Ávila, 47010 Valladolid, Spain

**Keywords:** social inclusion, rights, self-determination, people with disabilities, grassroots sports

## Abstract

Over the past decade, a growing body of evidence has highlighted the positive impact of full and equal participation in sport on the quality of life (QoL) of people with intellectual disabilities (IDs). However, access to mainstream sports remains limited due to persistent barriers, which leads to low participation levels among people with IDs. In this context, the Mixed Ability (MA) model offers an innovative approach by promoting the inclusion of people with and without disabilities in the same mainstream teams, without classification processes or modifications to the rules of the sport. Therefore, this study aimed to explore the relationship between rugby MA and the QoL and the needs of players with IDs. Using a convergent mixed methods design, the quantitative aspect involved 46 participants aged 18–57, engaged in eight different rugby teams, while the qualitative component included a focus group with four ID players from a rugby team. The findings revealed a positive correlation between higher QoL and the duration of participation in mainstream sports among the participants. Furthermore, a positive trend was also observed among individuals with moderate and severe intellectual disabilities. Qualitatively, participants highlighted the model’s emphasis on inclusivity, familial bonds, enjoyment, community spirit, active engagement, and opportunities to experience new activities. The shift towards inclusion requires modifying the community so that every person has opportunities to lead a valuable, meaningful, and self-determined life. In this sense, the MA sports model emerges as a potent instrument for fostering inclusive communities and enhancing the QoL of people with IDs.

## 1. Introduction

“Intellectual disability (ID) is characterized by significant limitations both in intellectual functioning and adaptive behavior as expressed in conceptual, social, and practical adaptive skills” [1]. People with IDs have the same right to be included in the community and to live independently, with equal options as others to decide with whom and how to live. However, their participation rates in sports are lower compared to people without disabilities [2]. Many people with IDs live segregated and lack control over their daily lives, particularly those living in institutions. This is mainly due to the insufficient provision of adequate community-based services [3].

The 2030 Agenda of the United Nations (UN) includes reducing inequalities within and among countries as one of its Sustainable Development Goals. Moreover, Article 30 of the Convention on the Rights of Persons with Disabilities (CRPD) establishes that participation in sports should be a right for all people on equal terms, allowing people with disabilities (PwDs) to participate in sports at all levels [4]. The principles and values included in the CRPD are linked to the concept of quality of life (QoL) [5]. The most recent definition of QoL provided by Schalock et al. [6] is interpreted as a multidimensional phenomenon composed of eight fundamental dimensions: self-determination (SD), rights (RI), emotional well-being (EW), social inclusion (SI), personal development (PD), interpersonal relationships (IR), material well-being (MW), and physical well-being (PW), influenced by personal characteristics and environmental factors [6]. This paradigm includes universal and cultural factors, with both subjective and objective components, where personal perception is key to QoL [7]. In this regard, community participation is an essential dimension of human functioning and a central element in the QoL of PwDs [8,9].

Over the past decade, it has been reflected that full and equal participation through sports is important, as it strengthens the social ties of people with IDs, fostering their social contacts, interactions, and connections, as well as helping to establish networks and friendships [10,11]. This, in turn, promotes positive attitudinal changes toward disabilities in society [12]. Furthermore, sports participation is associated with improved psychological well-being, higher self-esteem, and a reduced risk of anxiety and depression [11,13]. Notably, team sports offer greater benefits than individual sports, primarily due to the stronger social interactions and psychological support inherent in team-based activities [14]. Therefore, sport clubs can be considered a key tool for building inclusive communities and contributing to social well-being [15].

In the Spanish context, Law 39/2022 on Sport [16] reinforces the promotion of inclusive sport as a practice that fosters joint participation between people with and without disabilities on equal terms. However, physical activity often declines during young adulthood, as individuals both with and without disabilities move away from the structured routines of school and adopt more sedentary lifestyles [17]. In this sense, various barriers hinder their participation in mainstream sports, leading to higher levels of physical inactivity [11,18,19,20]. Opportunities to participate in inclusive sports groups remain scarce or, in many cases, non-existent [21]. As a result, sports for PwDs are often delivered in segregated formats, which reinforces patterns of exclusion and discrimination within mainstream sports [22]. Critics such as Navas et al. [23] argue that the pandemic has highlighted the detrimental effects of segregated approaches. Accordingly, offering segregated sports options contributes to maintaining a societal norm that treats the separation of disabled individuals as something normal and acceptable [24]. For this reason, organizations are responsible for promoting necessary change processes to eliminate passive and depersonalized approaches. Replacing the historical paradigm that prioritizes defectology, segregation, devaluation of individuals, and institution-based services with a community-based approach focused on QoL and individualized supports is essential [25]. Consequently, it is essential to foster communities where PwDs can choose to participate in any sport in the way they want [26]. Notwithstanding, when community initiatives are shaped by groups that are largely uniform—often White, middle-class, and non-disabled—they risk overlooking the varied needs and experiences within the wider population [27]. Therefore, the involvement of PwDs in the planning processes enhances the understanding of their abilities and promotes the development of more accessible and inclusive sports programs [28].

In this context, the Mixed Ability (MA) model emerged organically by PwDs in response to the absence of inclusive opportunities for them to play full-contact rugby. International Mixed Ability Sports (IMAS) formalized this approach, which has since evolved into a social movement promoting inclusivity within mainstream sports [29]. MA teams are often embedded within well-established sports clubs, functioning as just another team within the wider club structure. Nowadays, a wide range of sports are played under the MA model, including rugby, cricket, floor curling, football, golf, tennis, basketball, boxing, and more. MA teams can be found in countries such as the UK, Spain, Canada, Argentina, Chile, Ecuador, and Italy, among others. In Spain, rugby is the most developed MA sport, with a well-established structure and the largest number of active teams [30]. This model encourages people with and without disabilities to be part of the game without making adaptations to sports rules and without using classification or identification systems. Moreover, for its success, it is essential that athletes without disabilities understand that they are team partners and not volunteers. The more experienced players, both with and without disabilities, are responsible for providing support to the rest of the team. This fact helps reinforce the idea that there is no “them” and “us” to differentiate between people with and without disabilities, which is common in sports environments involving PwDs [31].

Unlike traditional segregated approaches, such as the Paralympic Games or Special Olympics, which focus on athletic excellence or structured recreational participation exclusively for PwDs, the MA model rejects separation and promotes full membership in community sports. While these segregated models have provided visibility and empowerment for some athletes, they often reinforce the idea that PwDs belong in “separate” sporting spaces and rely heavily on classification systems that limit autonomy and agency [32,33]. Unified Sports, promoted by the Special Olympics, involve teams composed exclusively of athletes with and without IDs; these teams are usually formed based on similar levels of ability, and competition categories are established accordingly [34]. In contrast, MA promotes equitable participation by removing distinctions, encouraging all participants to play and train together under the same conditions, rules, and expectations, thereby aligning more closely with the principles of SI, SD, and shared ownership of the sport [31].

An important aspect of this model is the SD of participants with disabilities, as they choose to take risks on their own [29]. To date, the United Kingdom and Italy are the only countries where empirical studies have been conducted to assess the outcomes of this model [29,31]. The impacts have been identified at three levels: (1) the individual level, with benefits for physical health, mental well-being, self-confidence, and sense of belonging; (2) the club level, with changes towards a more inclusive culture, attracting new members, more accessible infrastructure, and the development of professionals; and (3) the societal level, with changes in perceptions towards disabilities and a greater awareness of potential barriers to participation [31].

Given that the social inclusion of people with IDs in mainstream sports clubs remains an underexplored area, and in light of the limited research addressing their participation in inclusive community-based settings in the Spanish context, it is essential to examine both the feasibility and effectiveness of such practices [17,35]. Accordingly, the objectives of this research focused on (1) identifying QoL considering the eight dimensions proposed by Schalock & Verdugo [36] of MA sports players with IDs, (2) analyzing the relationship between MA sports and QoL, and (3) determining the opinions and needs of people practicing MA sports. The study hypothesized that (1) the number of years of participation and frequency of involvement in MA rugby would be positively associated with improvements in QoL among people with IDs, and (2) participants would describe MA rugby as an inclusive and empowering community sport that promotes personal growth, social belonging, and emotional well-being.

## 2. Methodology

### 2.1. Study Type and Design

This study employed a mixed methodology with a convergent design. A triangulation model was adopted, which involves the simultaneous collection of quantitative and qualitative data and the integration of both types of data to achieve a deeper understanding of the research problem [37]. This approach allows leveraging the strengths of both methods and resolving possible inconsistencies between qualitative and quantitative analyses [38].

### 2.2. Participants and Procedure

First, participants were selected based on the following inclusion criteria: being over 18 years of age, being identified as having an ID, and being active members of MA rugby teams previously contacted by the research team. Regarding exclusion criteria, no formal exclusions were established beyond not meeting the inclusion criteria. However, individuals who did not provide informed consent were not included in the final sample. Participant selection was conducted through non-probability convenience sampling to ensure compliance with the study’s specific requirements. All prospective participants received detailed information about the purpose of the study, the procedures involved, and the ethical guarantees, including confidentiality and the voluntary nature of participation. To ensure informed participation, all explanations regarding the study were provided using accessible and adapted communication strategies suitable for people with IDs. This included the use of plain language, visual supports when needed, and the possibility of involving support people in the process. In addition, coaches from the MA teams were previously informed and trained about the study’s objectives and procedures, so they could help explain the information clearly to both the participants and their support networks.

The recruitment process for the quantitative phase was carried out in collaboration with IMAS. Coaches from various MA teams, including Gaztedi, Arrasate, URA clan, Hernani, Incluindus, Salvador, Crat-sharks, and Iruña, were contacted to facilitate the distribution of invitations to the players to participate in the study. A total of 46 participants voluntarily chose to take part in the study. Prior to data collection, the researchers contacted the coaches to inform them about how families could support participants in completing the questionnaires. Prior to data collection, the researchers contacted the coaches to inform them about how families or trusted individuals could support participants in completing the questionnaires. Support was provided by people close to the participants, such as family members or support workers, who were familiar with their communication styles and daily routines. These supporters were instructed to avoid influencing the participants’ answers, ensuring that responses reflected their own views. This support included helping with reading and understanding the questions, navigating the online platform, providing assistance in a non-intrusive manner, and ensuring that participants’ responses remained as authentic and self-directed as possible, while still accommodating their individual support needs.

All participants provided informed consent, in accordance with the current regulations on privacy and personal data protection. Quantitative data collection was conducted through a questionnaire distributed via the Google Forms^®^ platform, which facilitated both the distribution and data collection processes. The questionnaire was sent directly to participants’ personal email addresses and/or phone numbers, as provided by the team coaches. The deadline for completing the questionnaire was set for two weeks, with an additional reminder sent out until the end of a one-month period.

For qualitative data collection, semi-structured interviews in a focus group format were used. This approach allowed for greater flexibility in exploring key topics or clarifying any confusing information that arose during the interview. The Incluindus MA club was contacted to invite all players to participate voluntarily in the interviews. This team was selected due to its well-established engagement with the MA. All players were invited to participate on a voluntary basis through trusted intermediaries who had no hierarchical relationship with the participants. This ensured a safe and pressure-free environment for recruitment. Ultimately, four players chose to take part, and interviews were conducted following a consistent guide while allowing space for follow-up questions and clarification. Interview guidelines were followed to maintain consistency across all sessions while allowing for follow-up questions to clarify important points that emerged.

### 2.3. Instruments

A sociodemographic questionnaire was conducted to collect information on age, gender, ID degree, MA team name, years played in MA, the number of times they saw the team, and the desire to see the team again. The classification of participants according to their degree of ID (mild, moderate, severe, or profound) was based on self-reports provided by the participants during the process.

In the Spanish context, there are several instruments to assess QoL. However, most only involve the report of others and are not specifically aimed at people with IDs. Therefore, the Inico-Feaps scale was used, which assesses the eight dimensions of QoL in people with IDs over 18 years old. It consists of 72 items distributed in each dimension with a four-point Likert response format. This scale is freely available for use in research and practice [39].

For qualitative data acquisition, a script composed of 12 questions linking each with the eight dimensions of the QoL paradigm [7] was used. This was evaluated by an expert committee consisting of two people from the University Institute of Community Integration (INICO). In addition, the script was pilot tested with two people with IDs to ensure the clarity, accessibility, and appropriateness of the questions. Some examples of guided questions were: “*How do you feel when playing Mixed Ability rugby?*”, “*How do you participate in decisions made during the game?*”, and “*How and by whom do you receive help or support?*” With the aim of exploring a specific set of questions related to MA and QoL. The questions were formulated openly and broadly, aimed at exploring participants’ experiences and perceptions.

### 2.4. Data Analysis

Quantitative statistical analyses were performed using the IBM SPSS Statistics 19.0 software for Windows. Descriptive statistics were conducted for the independent variable age (maximum, minimum, and mean) and frequency statistics for the independent variables gender, degree of ID, MA team name, years played in MA, the number of times they see the team, and the desire to see the team again, as well as for the dependent variables QoL, SD, RI, EW, SI, PD, IR, MW, and PW. For hypothesis testing, an average of the scores obtained by the participants was performed, as well as for each of their dimensions. Moreover, to assess the internal consistency of each subscale, Cronbach’s alpha coefficients were calculated.

For the inferential analysis, a multiple linear regression was performed to examine the extent to which several predictor variables (age, gender, intellectual disability, number of team members, years of participation in MA, level of participation, and intention to return to the team) explained the variance in the overall QoL score. Prior to conducting the regression, key statistical assumptions were tested. Normality was assessed through histograms, Q–Q plots, and the Kolmogorov–Smirnov test; linearity and homoscedasticity were checked using residual plots; and multicollinearity was examined through tolerance and variance inflation factor values. All analyses were performed by setting the critical significance level at 0.05. In addition, to assess the adequacy of the sample size, a post hoc power analysis was conducted using G*Power (version 3.1.9.7) based on the R^2^ obtained from the regression model.

On the other hand, qualitative data analyses were conducted using the Nvivo 15 software package. After conducting and reading the verbatim transcriptions, a categorical system was developed, which included coding in the meta-categories of QoL, beliefs, and needs about MA and the frequency of participation. These codes were grouped into sub-themes that better captured most of the codes assigned in response to the topics discussed during the interview. To ensure the reliability and rigor of the thematic analysis, the process was conducted independently by authors one and three. Both researchers familiarized themselves thoroughly with the data and generated initial codes separately. These codes were then compared and discussed to identify convergences and divergences. In cases of disagreement, discrepancies were resolved through consultation with the last author, ensuring consensus and consistency throughout the coding process.

### 2.5. Ethics Committee

Regarding data handling and ethical considerations, all collected information was processed in accordance with privacy regulations, ensuring the anonymization of the responses and the protection of personal information. The data were securely stored, with access restricted exclusively to the research team.

In addition, approval was obtained from the Ethics Committee of the Pontifical University of Salamanca, as documented in Record No. 14/06/2024, prior to the commencement of the study, and any methodological changes were reported to the committee to ensure transparency and ethical compliance.

## 3. Results

### 3.1. Quantitative Results

The reliability of the questionnaire items was assessed, resulting in a Cronbach’s Alpha of 0.922, which demonstrates excellent internal consistency. The sample consisted of 46 rugby players, of whom 21.7% (n = 10) were women and 78.3% (n = 36) were men, representing teams such as Gaztedi, Arrasate, URA clan, Hernani, Incluindus, Salvador, Crat-sharks, and Iruña. Of the participants, 60.9% had a mild ID, 37% had a moderate ID, and 2.1% had a severe ID. The average participant age was 30 years, with an age range of 18–57. Regarding experience in MA sports, the length of practice varied: 17.4% had less than 1 year, 21.7% had 1 year, another 21.7% had 3 years, 10.9% had 4 years, and 28.3% had 5 years or more.

A standardized QoL average of the participants (M = 81.8) was obtained, as well as for each of their dimensions: SD (M = 10.73), RI (M = 10.95), EW (M = 9.65), SI (M = 10.43), PD (M = 9.17), IR (M = 9.97), MW (M = 10.97), and PW (M = 9.89). This places the study participants, according to the scale used, in a QoL index of 102, equivalent to a median percentile of 55, as well as for the dimensions of, in SD, a percentile of 63, in RI, a percentile of 63, in EW, a percentile of 50, in SI, a percentile of 50, in PD, a percentile of 40, in IR, a percentile of 50, in MW, a percentile of 63, and, in PW, a percentile of 50. Through these data, we can identify that the participants’ greatest needs were in the PD dimension (Table 1).

However, it is important to note that 50% always and 30.4% frequently reported “learning things that make them more independent”. Similarly, 52.2% always and 26.1% frequently indicated that “they have opportunities to learn new things”. Regarding the MA model, 58.7% of the participants declared that, once they went home, they were very eager to see the team again (34.8% stated they were eager, and the rest showed indifference). However, 63% only saw the team members once a week and 34.8% twice a week.

Furthermore, a regression analysis was conducted to evaluate the relationship between the variables associated with MA implementation (team, years played, intensity of participation, and desire to see the team again) and other independent variables (age, gender, and degree of ID) that could predict QoL (F (7,38) = 2.93; *p* = 0.015). It was found that this model explains 35% of the variation in the sample’s QoL (R^2^ = 0.35). The power analysis indicated a statistical power of 93.7% (f^2^ = 0.538; α = 0.05; N = 46; seven predictors), supporting the adequacy of the sample size. This reflects a high probability of detecting true effects, thereby strengthening the validity of the regression results.

Moreover, the analysis revealed that the only MA-associated variable that was significant in predicting QoL was the number of years participants had been playing MA (B = 0.379, 95% CI [0.829–5.835], *p*= 0.01) (see Table 2).

**Figure 1:**
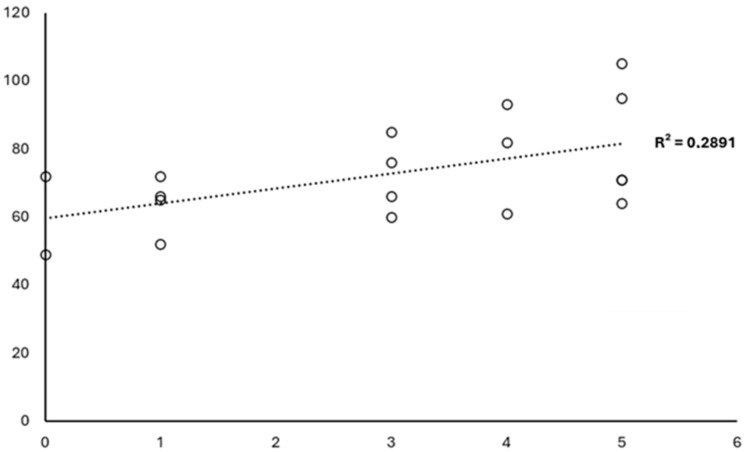
Regression of the variable quality of life with respect to the variable years played in individuals with moderate and severe intellectual disabilities.

Additionally, the influence of other variables unrelated to the MA model on participants’ QoL was highlighted. The degree of ID was found to be significant in predicting QoL (B = −15.294, 95% CI [−24.178 to −6.441], *p* = 0.001). This finding indicates that, after controlling for the other variables in the model, participants with a higher ID degree reported significantly lower levels of QoL. The standardized beta coefficient also highlights that this variable had the strongest effect size among all the predictors included. Consequently, a regression analysis excluding those with a mild degree of ID was conducted, therefore selecting those with moderate and severe IDs, once again finding a positive trend between the years played and QoL (B = 0.511, 95% CI [47.851–73.216], *p* = 0.030) (Figure 1).

### 3.2. Qualitative Results

The focus group participants were three male players and one female player with IDs from the MA Incluindus team. One participant, aged 28, had a mild ID and more than five years of experience in the sport. Two participants had moderate IDs: one aged 24 with less than one year of experience and another aged 23 with just over one year of experience. The fourth participant, aged 24, had a severe ID and more than five years of rugby experience.

The analysis of the transcriptions allowed identifying the most important discussion topics that repeatedly appeared in the dialogues. In the word frequency analysis based on the coding, certain dimensions reveal more pronounced differences in their mention rates, such as SI, IR, PD, and SD. The participants indicated that the MA sport had a direct impact on improving their lives. This was expressed in opinions such as “MA has changed our lives compared to before” or “it has changed my life, MA helped me to know other things, meet new people, new experiences”.

Opinions about MA tended to be inclusive, familiar, fun, community-oriented, participatory, and where there was an opportunity to experience new things. Additionally, they highlighted that, within the MA context, they were the main actors of their lives, emphasizing how the community environment had provided them, in a self-determined manner, the opportunity to make decisions and set meaningful personal goals within the club. Table 3 presents the most notable comments, highlighting participants’ opinions and needs regarding the eight dimensions of playing MA.

#### 3.2.1. Social Inclusion

Participants consistently described MA as a sport environment that fosters community and inclusion, contrasting it with previous experiences in segregated or conventional sports settings. They expressed that they were not treated differently, nor were specific adaptations made for their participation—something they interpreted positively, as it reinforced their sense of equality within the team dynamic. The sport was perceived as “normal rugby” where every player, regardless of ability, shared the same space, rules, and objectives. Moreover, participants valued being recognized as full members of the team. The sense of belonging extended beyond the pitch, reflecting a broader feeling of being part of a community that values diversity and inclusion.

This inclusive structure contributed to a deeper sense of social integration. One participant shared “Yes, I would like to spend more time with the team”, revealing a genuine desire for more frequent and meaningful interactions beyond the scheduled sport activities. Although most comments reflected strong group cohesion, such statements highlight the need to strengthen informal social opportunities that may foster friendships and peer support outside of training sessions or matches.

#### 3.2.2. Rights

The transition from segregated environments to inclusive sport settings was described as a powerful and liberating experience. Participants highlighted the significance of being treated equally and fairly without being defined by their disability. They described MA as a space where they felt heard, respected, and valued as individuals.

One of the most consistent themes was the recognition of their right to participate in sports under the same conditions as everyone else. For many, this was the first time they had felt truly included in a team where ability did not determine hierarchy or access. The emphasis on equality and mutual respect contributed to a positive redefinition of their identity as athletes. Several players mentioned that, in MA, new members were always welcomed without judgment and that the culture of the club encouraged respect and inclusion from day one. This environment was crucial in counteracting past experiences of exclusion and marginalization, and it offered a tangible example of the realization of RI in practice. One participant stated “They give me the opportunity”.

#### 3.2.3. Personal Development and Self-Determination

Involvement in MA was also linked to personal growth and the development of new competencies. Players reported that participation in the sport enabled them to learn and improve various skills, not only technical or physical but also social and emotional. This skill acquisition was frequently mentioned as a source of pride and confidence, and many participants associated it with a boost in self-esteem.

A key element that emerged was the perception of autonomy and active contribution. Participants did not feel like passive beneficiaries; instead, they emphasized the role they played in helping others. When new members joined the team, players with ID often supported their integration, regardless of the newcomers’ abilities. This dynamic was especially meaningful, as it demonstrated the bidirectional nature of support within the team and reinforced feelings of competence and self-worth among all the players. Such experiences align with the QoL domain of SD, as participants felt they had agency and responsibility within the team, contributing actively rather than being merely accommodated. One player argued “When we are on the field we do decide”.

#### 3.2.4. Interpersonal Relationships

IR emerged as a central theme in participants’ narratives, reflecting the profound social value of their involvement in MA. For many, the team environment provided an opportunity to build meaningful connections. Participants described a strong sense of camaraderie, noting that MA fostered authentic relationships. Teamwork was frequently highlighted as a core element of the experience. As one participant explained, the sport was played “in union with the team as if it were another ordinary one”, indicating that their relationships were not shaped by a disability label but rather by shared goals and mutual engagement on equal terms. This collaborative approach helped to break down social barriers and build inclusive bonds within the group.

Moreover, MA was seen as a space where supportive interactions were not only received but also offered. Several players shared how they were able to assist teammates—regardless of whether they had a disability or not—emphasizing the reciprocal nature of relationships. This active exchange of support contributed to feelings of belonging and recognition, reinforcing their role not only as participants but as valuable contributors to the group dynamic.

However, while the overall quality of relationships was described positively, some comments suggested room for growth. For instance, one participant expressed a desire to “spend more time with the team”, implying that social interactions outside of training sessions could be limited. The desire to deepen bonds beyond structured sport contexts suggests that, while a sense of belonging exists, opportunities for organic SI remain uneven. This points to the potential for enhancing informal social opportunities, such as shared meals, events, or activities beyond the sport, which may further strengthen interpersonal ties and contribute to a richer community experience.

#### 3.2.5. Well-Being

The interviews revealed that the MA rugby experience contributed positively to participants’ overall well-being, encompassing physical, emotional, and, to a lesser extent, material dimensions. From a FW perspective, players expressed satisfaction and pride in being able to engage in a sport they enjoyed, noting improvements in fitness, routine, and energy levels. Some commented on how being part of a team motivated them to stay active and maintain healthy habits over time, which they had not always achieved in previous sport or leisure contexts.

Emotionally, the MA environment appeared to offer a safe and motivating space, although some participants found it difficult to express their emotions openly within the group. While many reported feeling happy, accepted, and valued in the team, a few testimonies indicated emotional reservations. For instance, the quote “I keep my problems to myself (…), I share them with other people” suggests that, while the team setting fosters inclusion, some participants preferred to reserve more personal or emotionally sensitive disclosures for other trusted contexts, such as with family members or close friends. This indicates that, although the team was perceived as a supportive environment, it was not necessarily viewed as the primary space for managing or discussing emotional difficulties. Such distinctions point to the complexity of informal socialization processes in inclusive sports and highlight the importance of acknowledging individual preferences and comfort zones when fostering emotional openness within team settings. Despite these nuances, the overall atmosphere of respect and camaraderie supported participants’ emotional resilience and self-confidence, contributing to a broader sense of psychological well-being.

In terms of MW, while this aspect was not central to the interviews, some indirect insights emerged. One significant barrier mentioned was transportation. A participant explained “I have to take two buses and I get home around 11. I don’t have enough time; it’s like I have to make too many detours.” This statement reflects how economic and infrastructural conditions can affect access to sports and, by extension, QoL. The time, cost, and effort required to participate may pose challenges for individuals with fewer resources or limited support networks. Even so, participants remained committed, which speaks to the value they placed on the experience and the benefits they derived from it.

## 4. Discussion

This study aimed to evaluate the impact of the MA sports model on people with IDs. Quantitative results indicate that the QoL for individuals with IDs participating in MA sports is at a median percentile. However, high scores were observed in SD, RI, and SI. Qualitative analysis within the MA environment revealed improvements across all dimensions, with players describing their MA experience as inclusive, familiar, fun, community-oriented, and participatory.

The results partially supported the first hypothesis: a significant positive association was found between the number of years of participation in MA sports and higher QoL scores. However, no statistically significant relationship was observed between participation frequency and QoL. This suggests that sustained involvement over time may play a more critical role in perceived well-being than the short-term intensity of participation. On the other hand, the qualitative findings support the second hypothesis, indicating that participants perceive MA rugby as an inclusive and empowering sport that promotes personal growth, social belonging, and EW. Players reported feeling equal and respected within their teams, highlighting the value of being treated like any other athlete. They described the environment as one in which differences were normalized and their contributions valued, which, in turn, fostered PD, SD, and increased self-confidence. In this aspect, McCarron et al. [40] reported that the transition of people with IDs from an institution to the community was associated with better QoL. However, Sørensen and Kahrs [41] indicated that people with higher support needs often could not adopt the practices and values of mainstream sports. This often happens due to barriers such as a lack of programs, qualified coaches, and understanding toward people with IDs [42]. Moreover, social norms shape the extent to which PwDs can exercise SD in accessing and participating in sports, particularly through perceptions of competence, autonomy, and a sense of belonging. In this sense, ableism can directly undermine SD, and social practices within this context may serve to reinforce ableist attitudes [43]. In contrast, the quantitative section of this study found that people with moderate and severe IDs experienced a more positive QoL the longer they participated in MA. In addition, qualitative data show that participants emphasized the active role every player has in supporting others, reinforcing a sense of competence and mutual respect. The findings of Dyer & Sandford [31] indicated that the MA model could facilitate the participation and engagement of PwDs in mainstream sports. Therefore, observed increases in QoL may be attributed to strategies within MA clubs that focus on enhancing individual talents, encouraging participation, providing personalized support, and offering opportunities for personal growth [44]. Thus, Dyer et al. [27] highlighted that many sports clubs do not realize they are exclusionary until they are exposed to the MA model. In this sense, for those who fit the norm, normality often goes unnoticed. However, for those seen as different, it becomes more prominent and has a greater impact on how they experience society [45]. This raises an important question about whether people with higher support needs are truly offered meaningful choices and opportunities to participate or if their options are limited by structures that fail to recognize and support their autonomy, often shaped by ableist assumptions that they will not “fit in” under certain contexts. Such concerns underscore the importance of promoting a quality life through deinstitutionalization and from a non-ableist perspective, especially in a high-risk group such as those with higher support needs [3,23,26,40,43].

In this line, qualitative data revealed that participants emphasized how MA has positively transformed their lives, as they no longer face the segregation or stigmatization present in other clubs they previously joined. This is consistent with the findings of Hansen et al. [46], who reported that fears of bullying or exclusion discouraged people with IDs from participating in sports and physical activities. Participants also expressed that MA fosters an environment where PwDs also support their teammates, which may enhance decision-making, teamwork, and a sense of equality among participants. SI, in this context, extends beyond simply being present in the community; it also involves feeling accepted, having the power of choice, and playing a role in valued activities within it [47]. This inclusive environment appears to facilitate social connections, interactions, and the development of networks and friendships while also challenging societal perceptions of disabilities [27,29,31,48].

In addition, most participants demonstrated a strong or high desire to see the team again once they went home. Qualitatively, participants declared that IR were central, with many reporting strong team bonds and satisfaction within their teammates. According to Schlesinger & Nagel [49], clubs that offer adequate opportunities can act as catalysts for the stability of their members. However, many participants indicated that their interactions with teammates were generally limited to training sessions, and qualitative findings echoed this restricted social engagement. Several individuals expressed a desire for more frequent contact and reported feeling hesitant to share personal emotions. A study found that people with IDs struggle to transfer the social relationships formed through sports into the wider community [50]. In this sense, the frequency of contact is a key component in the structure of IR, and for SI to be truly effective, it must overlap with and mutually reinforce these relationships [51,52]. Therefore, we propose that, to fully leverage the benefits of MA, it may be necessary to increase the frequency of interactions to further strengthen social bonds and enhance the sense of community. Another barrier identified was transport. These results align with prior studies highlighting that limited access to safe and accessible transport poses a major barrier to the social participation and independent living of PwDs [28,53].

In addition, participants reported feeling safe during practice and highlighted the importance of maintaining the integrity of the sport’s rules. This perception supports Corazza and Dyer’s [29] view, which states that inclusive approaches create a safe space where players can take risks, make autonomous choices, and feel valued within the group. Furthermore, adjustments were described by participants as essential to enabling full participation without compromising the nature of the sport. In this sense, guaranteeing reasonable adjustments is essential to include individuals from diverse backgrounds and abilities and confront the deeply rooted ableism within institutional frameworks [54]. As such, social justice is imperative for achieving inclusive outcomes; therefore, allowing individuals to make their own choices is key to fostering a sense of belonging, equality, and empowerment [55]. According to the United Nations [4], participation in sports should be a right for all people on equal terms. This implies that it is essential for people with IDs to have the opportunity to participate in sports in the way they wish, with whom they wish to participate, and how they wish to participate [26]. Hence, it is fundamental to transition from practices that prioritize defectology, segregation, devaluation, and institution-based services to community-based approaches that align with QoL principles and provide individualized support [25].

### Limitations, Future Directions, and Practical Applications

This study has several limitations that should be taken into account when interpreting the findings. Firstly, the sample size was relatively small, which limits the generalizability of the results. This is directly related to the limited number of MA clubs in Spain, as this inclusive sport model is still in an early stage of development nationwide. Therefore, while the relationship between QoL and MA does not allow definitive conclusions, the observable trends are nonetheless noteworthy.

The use of non-probabilistic convenience sampling reduces control over external variables that could have influenced the results, such as differences in team structure, coaching styles, or regional support systems. Additionally, this sampling method limits the generalizability of the results. However, this approach was necessary due to the limited number of eligible participants and the specific characteristics of the study population. Identifying and recruiting a sufficient number of participants with IDs who voluntarily wanted to participate was particularly challenging. Despite these constraints, the sample represents a large proportion of the current people with IDs that practice MA in the country and provides valuable insights within this specific context. To obtain a more comprehensive understanding and validate these findings, future research should aim to include a larger number of participants, ideally selected through random sampling methods. Moreover, there could be a potential self-selection bias, as participation in the study was voluntary. This means that those who chose to take part may have had particularly positive experiences or strong opinions about MA, which may not fully represent the views of all team members.

Furthermore, the absence of longitudinal tracking restricts the ability to establish causal relationships between participation in MA rugby and improvements in QoL. Although the findings suggest meaningful associations, the cross-sectional design only captures a snapshot in time and cannot account for changes or developments over an extended period. To better understand the long-term effects of participation in MA sports on QoL, and due to the scarcity of longitudinal studies in the field, future research should employ mixed-longitudinal designs that track changes over time. Additionally, the originally planned in-person quantitative data collection had to be adjusted to an online format due to time and budget constraints. This format may have posed challenges for participants with diverse cognitive abilities. Although support was available when needed, completing the questionnaire digitally may have affected some people’s understanding or the accuracy of their responses. However, the coaches confirmed that the participants received support from family members and other close individuals during the process.

Practical applications of these findings can enhance the social value of sports and contribute to the creation of more inclusive and equitable communities. Sports organizations, policymakers, and associations of PwDs should work collaboratively to adopt inclusive models such as MA, promoting equal participation and shared experiences. Opportunities for informal social interaction beyond training should be encouraged to strengthen peer relationships. To address transport-related barriers, partnerships with local authorities or mobility support schemes should be explored. Moreover, communication materials and procedures should be made cognitively accessible, and people with IDs should be actively involved in decision-making processes to promote SD.

## 5. Conclusions

In conclusion, this study provides valuable insights into the impact of the MA sports model on the QoL of people with IDs. The findings suggest that participation in MA sports could enhance QoL, particularly in the areas of SD, RI, and SI. Quantitative results indicated that the longer individuals engage in MA sports, the higher their QoL tends to be. This suggests that sustained participation in inclusive and supportive environments could contribute positively to certain dimensions of QoL, although further research is needed to confirm causal relationships.

At the same time, the study also identified areas that warrant further attention. While participants described feeling accepted and equal within their teams, they also reported difficulties in building relationships beyond training sessions, including limited emotional sharing and logistical barriers such as transport. These context-specific limitations suggest that the social benefits of MA sports might be strengthened by increasing opportunities for interaction beyond the sport itself.

Overall, the study contributes to the growing body of research indicating that inclusive sport initiatives, when well-structured and responsive to individual needs, can promote more equitable and meaningful participation for people with intellectual disabilities.

## Figures and Tables

**Table 1:** Descriptive statistics of the quality of life dimensions.

Dimension	Minimum	Maximum	M	SD	α
FW	1.0	15.0	9.89	3.368	0.733
SD	1.0	16.0	10.73	3.275	0.839
RI	1.0	15.0	10.95	2.897	0.742
EW	2.0	15.0	9.65	2.899	0.731
SI	6.0	15.0	10.43	2.570	0.577
PD	1.0	15.0	9.17	3.233	0.759
IR	3.0	15.0	9.97	2.560	0.631
MW	3.0	14.0	10.97	2.534	0.649
Total CV	49.0	111.0	81.80	16.455	0.922

Note. Scores correspond to standardized values for each subscale and the total quality of life score.

**Table 2:** Multiple linear regression predicting the standardized quality of life.

Predictor	B	SE	β	t	*p*
Age	0.148	0.229	0.091	0.645	0.523
Gender	−2.089	5.353	−0.053	−0.390	0.698
ID degree	−15.294	4.388	−0.502	−3.485	0.001
MA team	0.781	0.525	0.212	1.490	0.145
Years in MA	3.332	1.236	0.379	2.695	0.010
Participation Level	1.006	4.434	0.033	0.227	0.822
Desire to see the team	−7.422	4.037	−0.281	−1.839	0.074

Note. Dependent variable: standardized quality of life. *p* < 0.05 is considered statistically significant.

**Table 3:** Relationship between the dimensions of QoL and the perceptions of participants in MA rugby.

Dimension	Perception of Participants
SD	“Without my family worrying about what time I get home” “We talk among ourselves and come to a joint decision” “Yes, I make my decisions” “And I say, please can we practice it” “Those who know less have a hard time, and we have to help them”
EW	“It’s okay, I feel safe playing MA” “Playing in a team is good, it gives us confidence” “We can share our emotions” “The MA environment is careful with me” “We receive supports” “I go to rugby to enjoy it to the fullest”
FW	“We feel safe playing this sport”
MW	“I have gone to Almería with the team” “We are very eager to participate in the IMART” “I would like to go not only to Cork but also to other places” “Thanks to Rugby they have found me a job”
RI	“The environment respects us” “It has changed my life because I can participate in a team sport. And what I played was segregated” “We have different supports”
PD	“Now I play in a team, something I didn’t do before. It was harder for me” “New things, which we have never done with others”
SI	“It is more community-oriented, more inclusive than other models” “MA is the same as playing normal rugby, but mixed” “There is inclusion if we all get together, if we separate it is not the same” “Inclusive sport should not have adaptations” “Anyone new is well received” “I went to basketball, football, and it is not the same. Here it is more fun, having fun is what matters most”
IR	“Even playing with the rival, it also feels like we are a big family, which is rugby” “In MA I work in a team” “It is like a union, it is like a family” “We communicate with each other”

## Data Availability

The data that support the findings of this study are available from the corresponding author, P.D.-F., upon reasonable request. The data are not publicly available due to privacy and ethical restrictions.
